# Supplementary figures and images for: Striking parallels between dorsoventral patterning in Drosophila and Gryllus reveal a complex evolutionary history behind a model gene regulatory network
Source: eLife. 2021 Mar 30;10:e68287. doi: 10.7554/eLife.68287 (PMC8051952; doi:10.7554/eLife.68287)

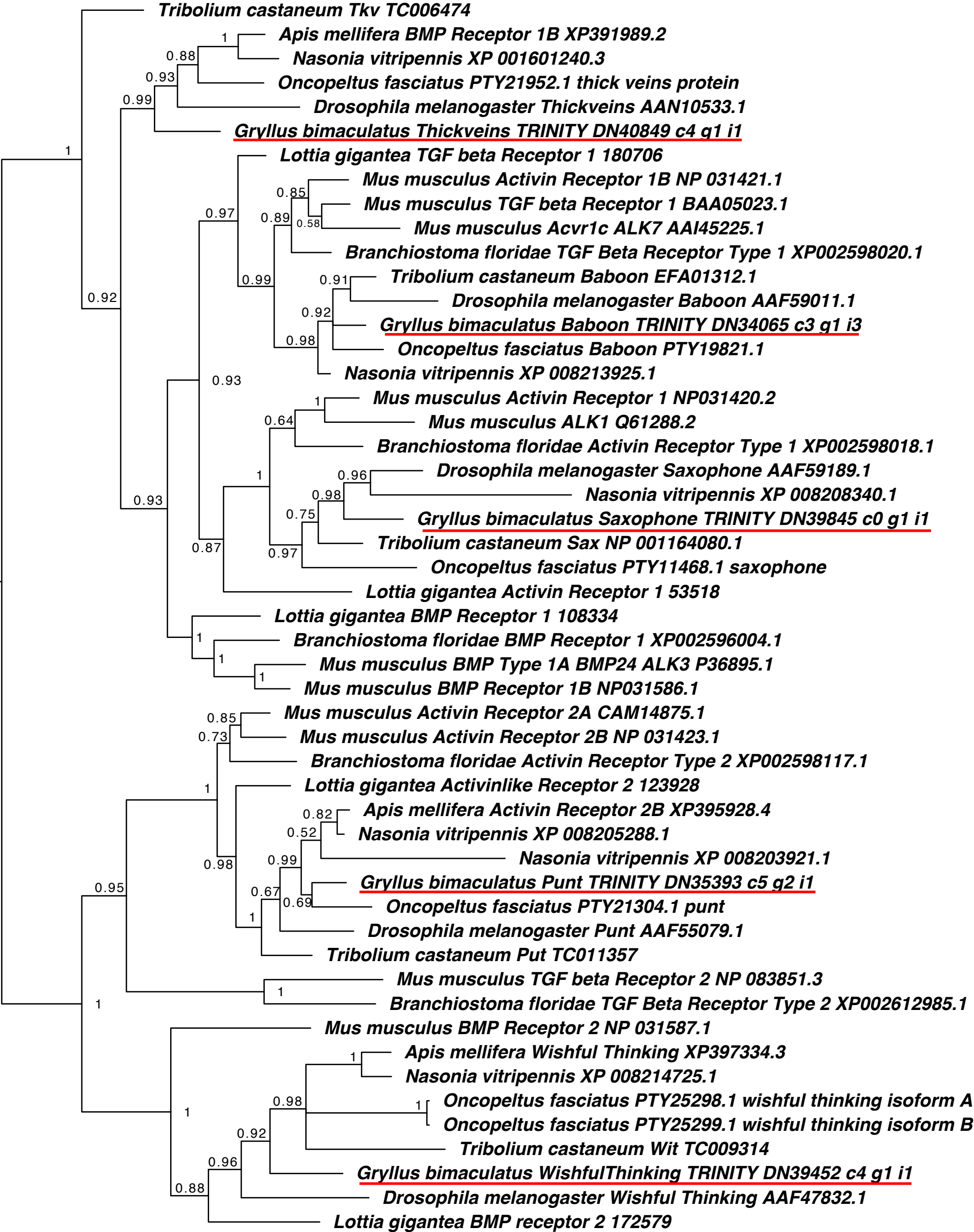

0.2

Supplement: Supplementary file 3. [file elife-68287-supp3.zip › File S3 /TreeFigures/ALKs.pdf]

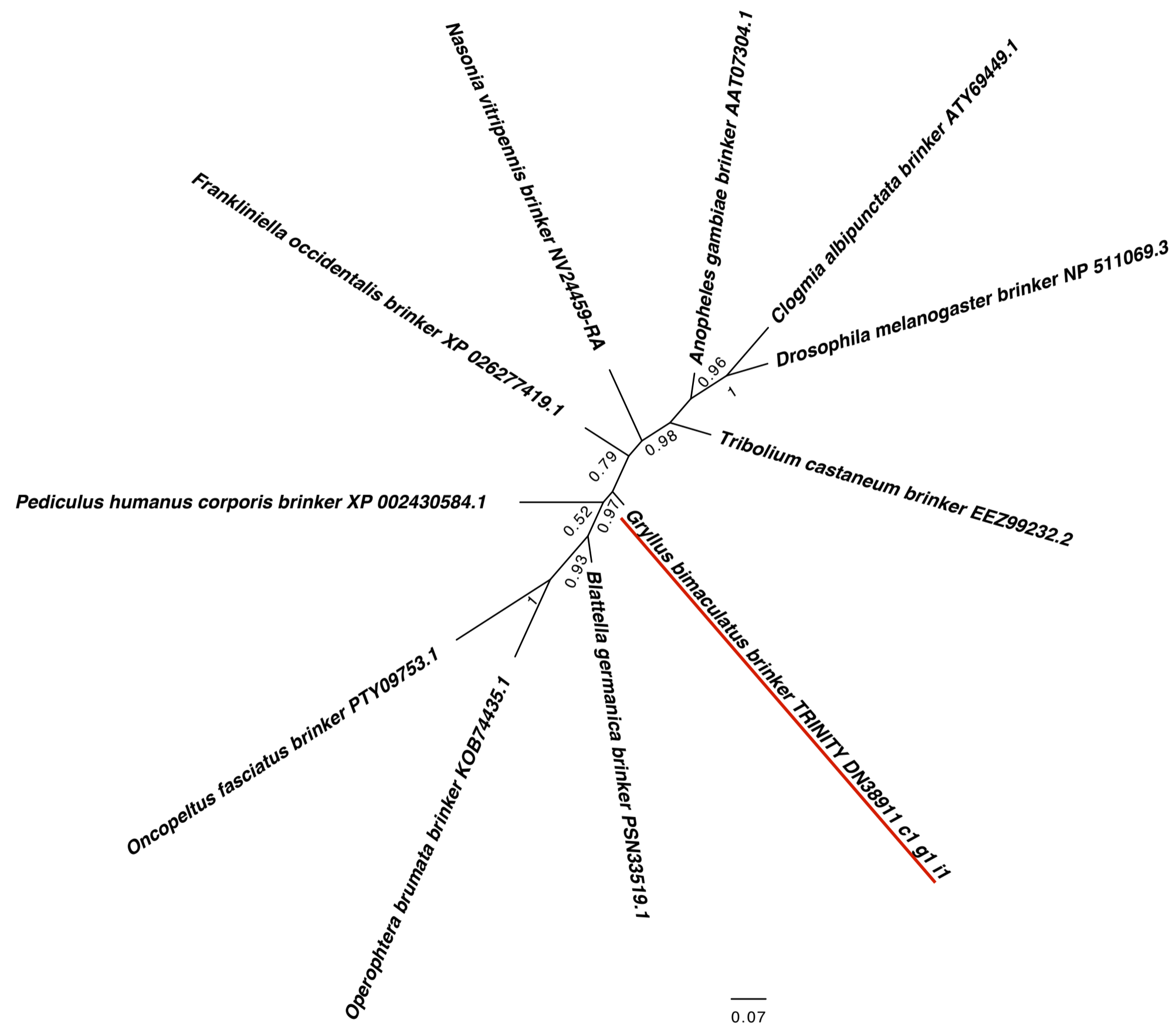

Supplement: Supplementary file 3. [file elife-68287-supp3.zip › File S3 /TreeFigures/brinker.pdf]

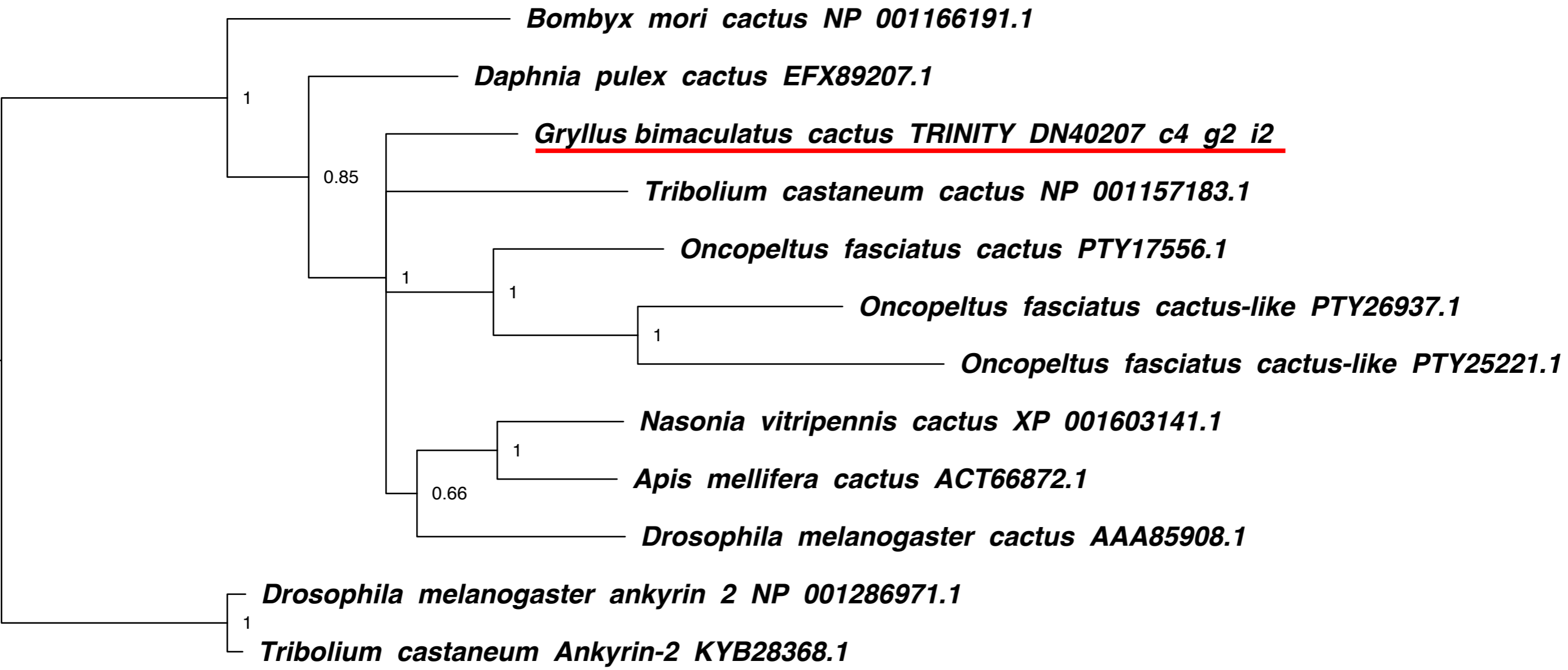

0.2

Supplement: Supplementary file 3. [file elife-68287-supp3.zip › File S3 /TreeFigures/cactus.pdf]

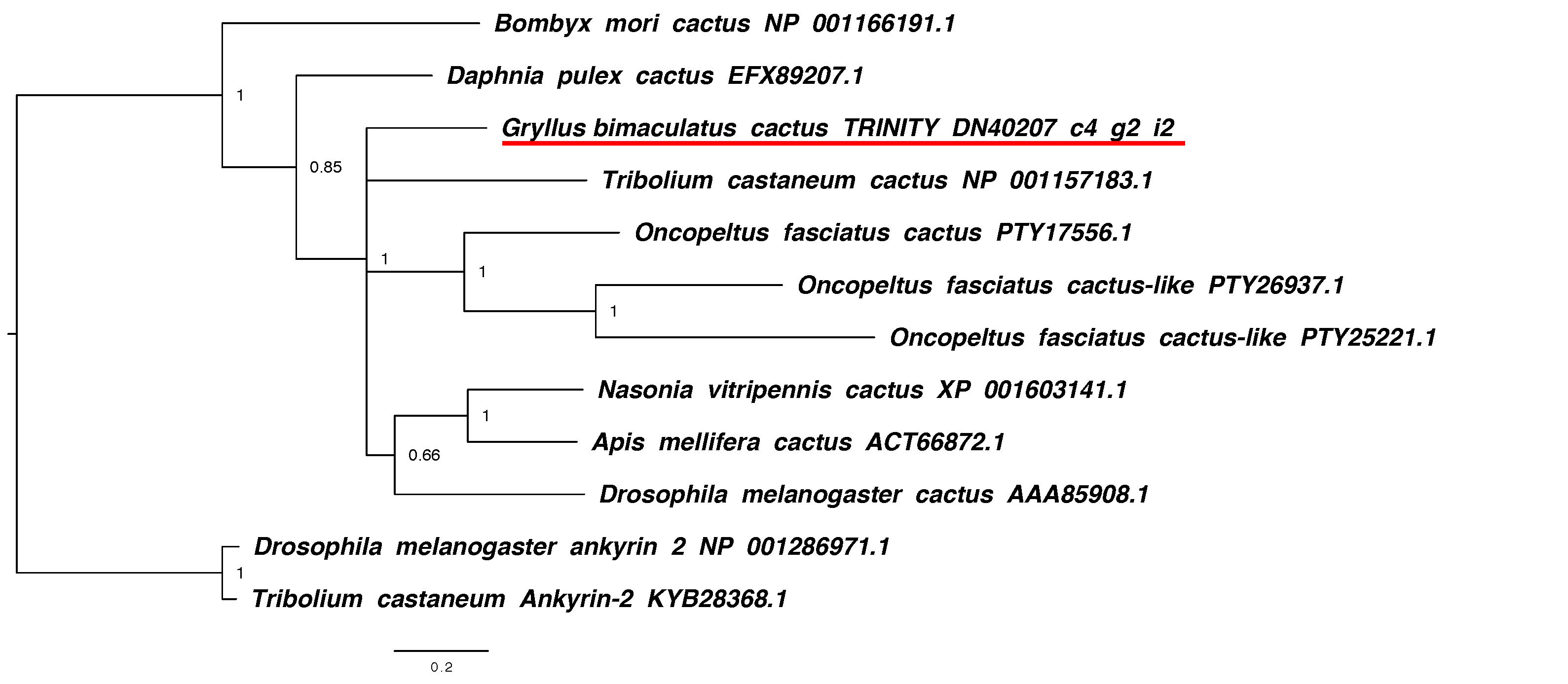

Supplement: Supplementary file 3. [file elife-68287-supp3.zip › File S3 /TreeFigures/cactus.png]

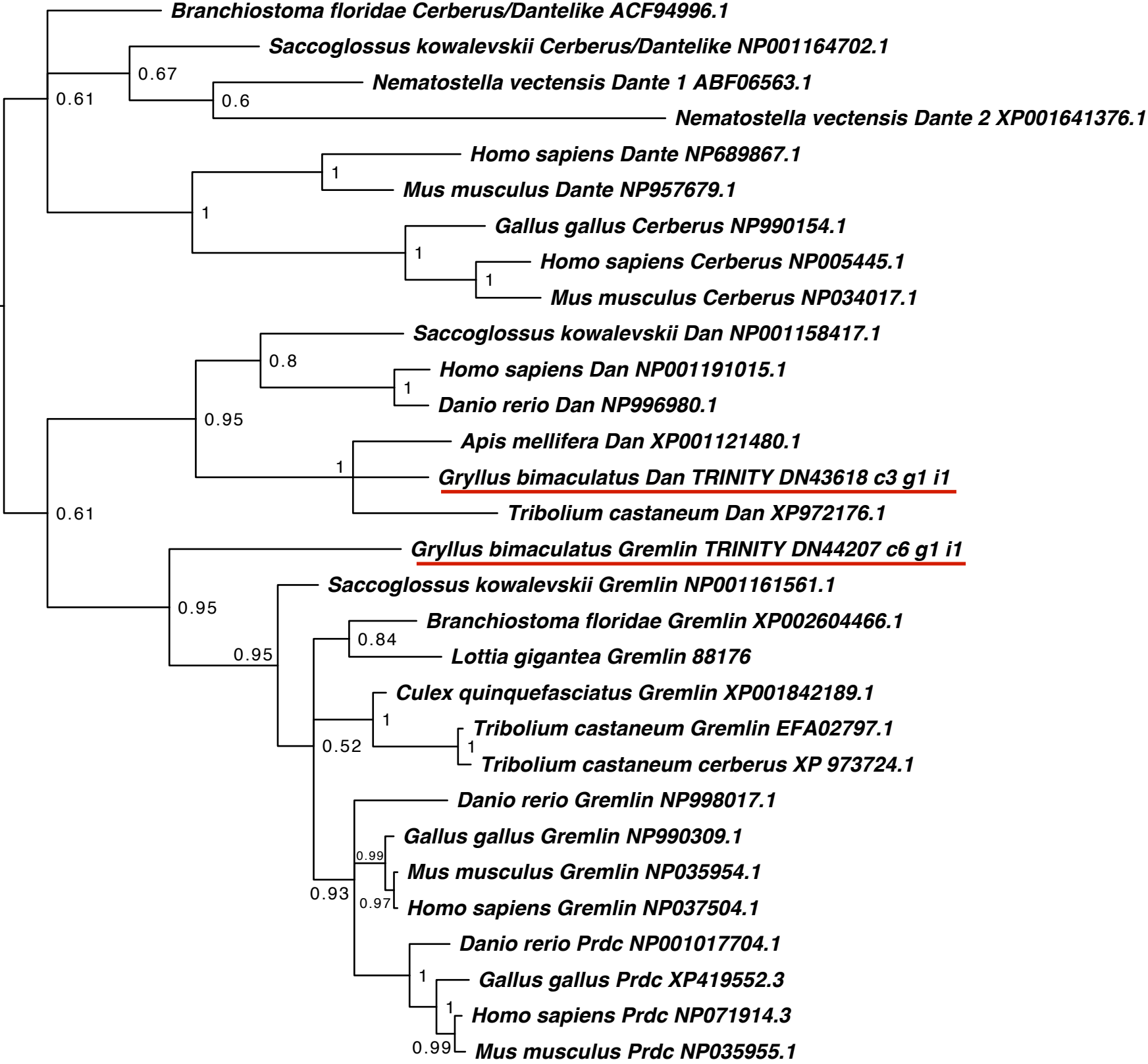

0.3

Supplement: Supplementary file 3. [file elife-68287-supp3.zip › File S3 /TreeFigures/Cerberus_Dan_Dante.pdf]

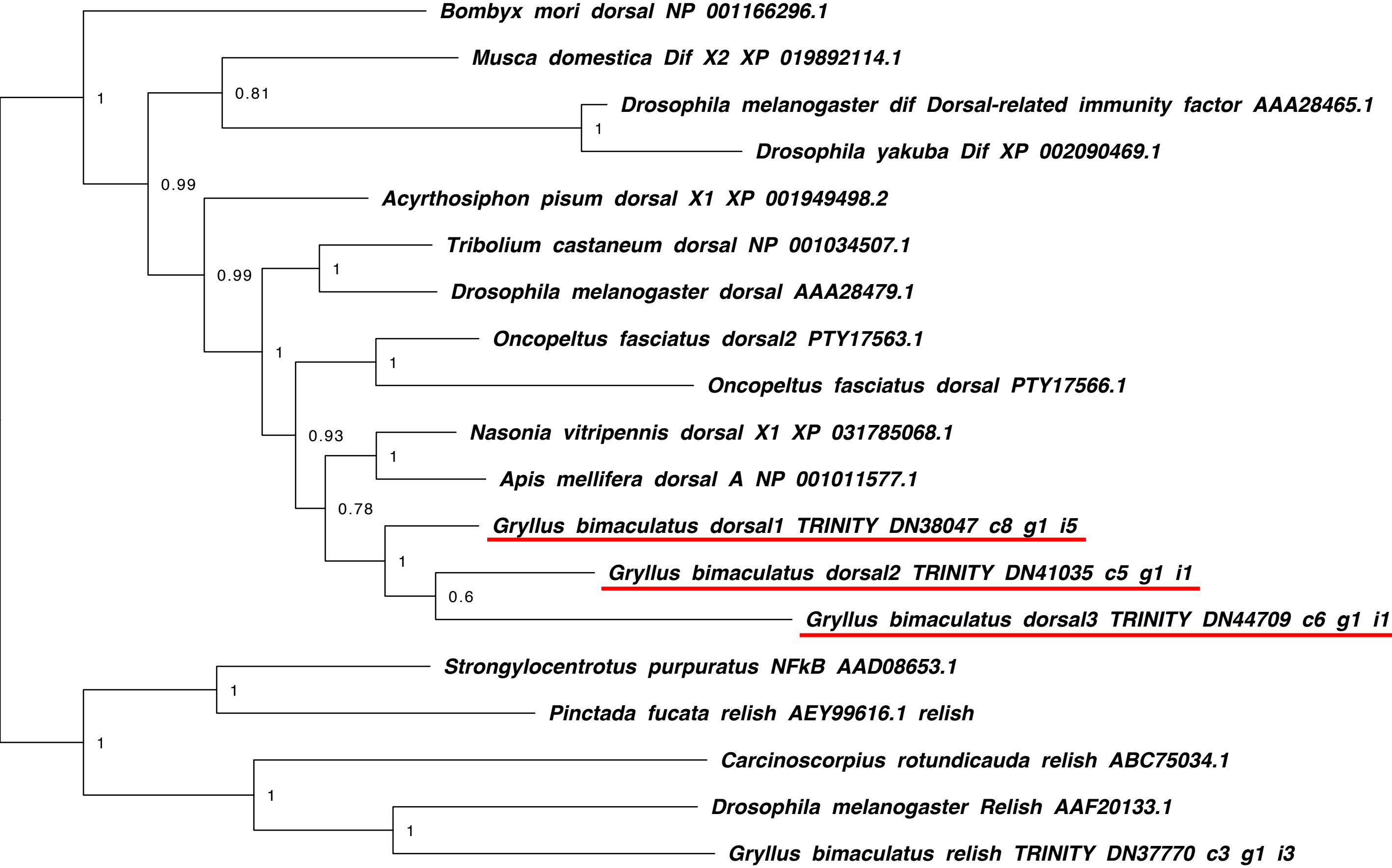

0.2

Supplement: Supplementary file 3. [file elife-68287-supp3.zip › File S3 /TreeFigures/dorsal.pdf]

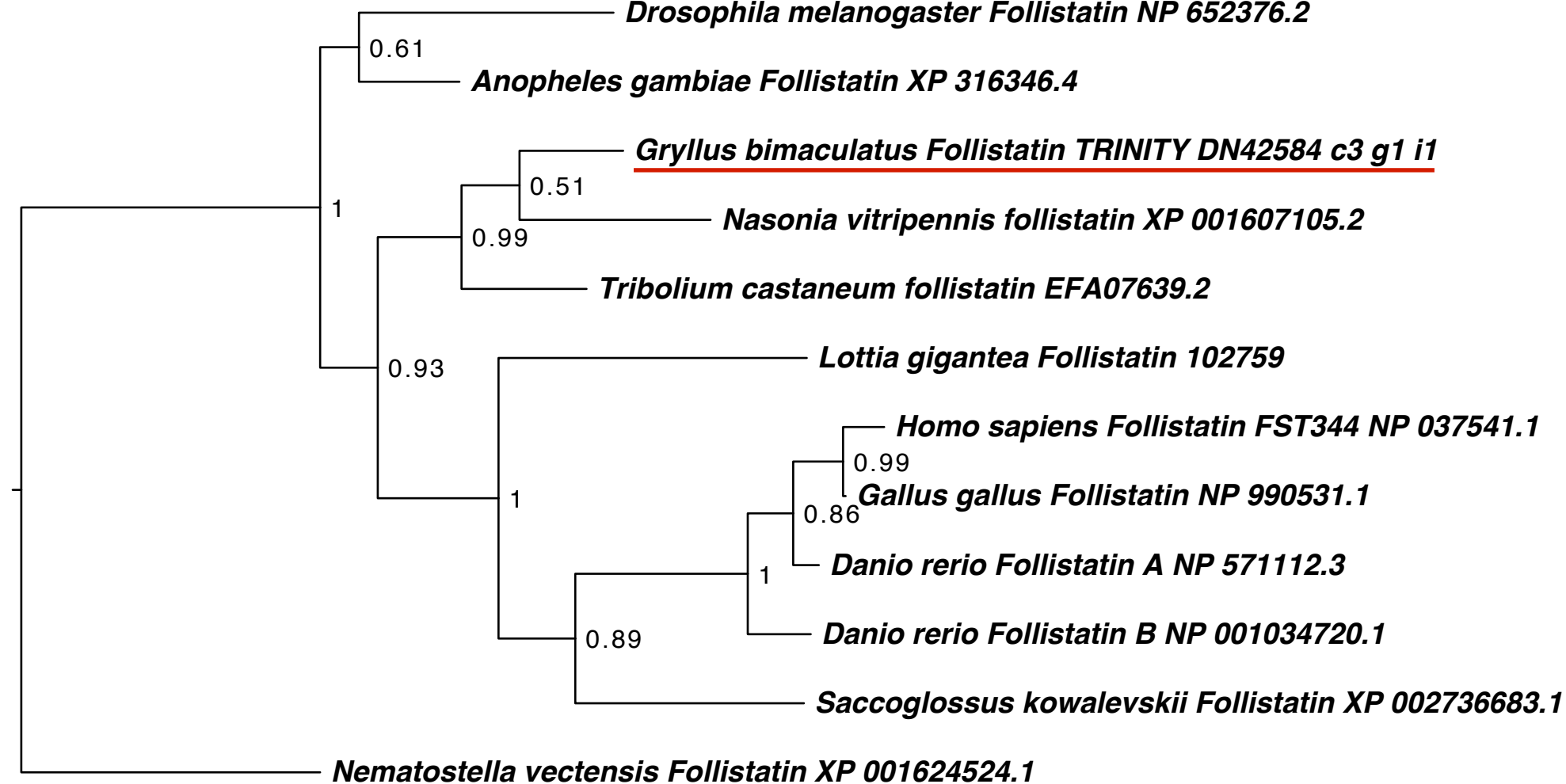

0.2

Supplement: Supplementary file 3. [file elife-68287-supp3.zip › File S3 /TreeFigures/follistatin.pdf]

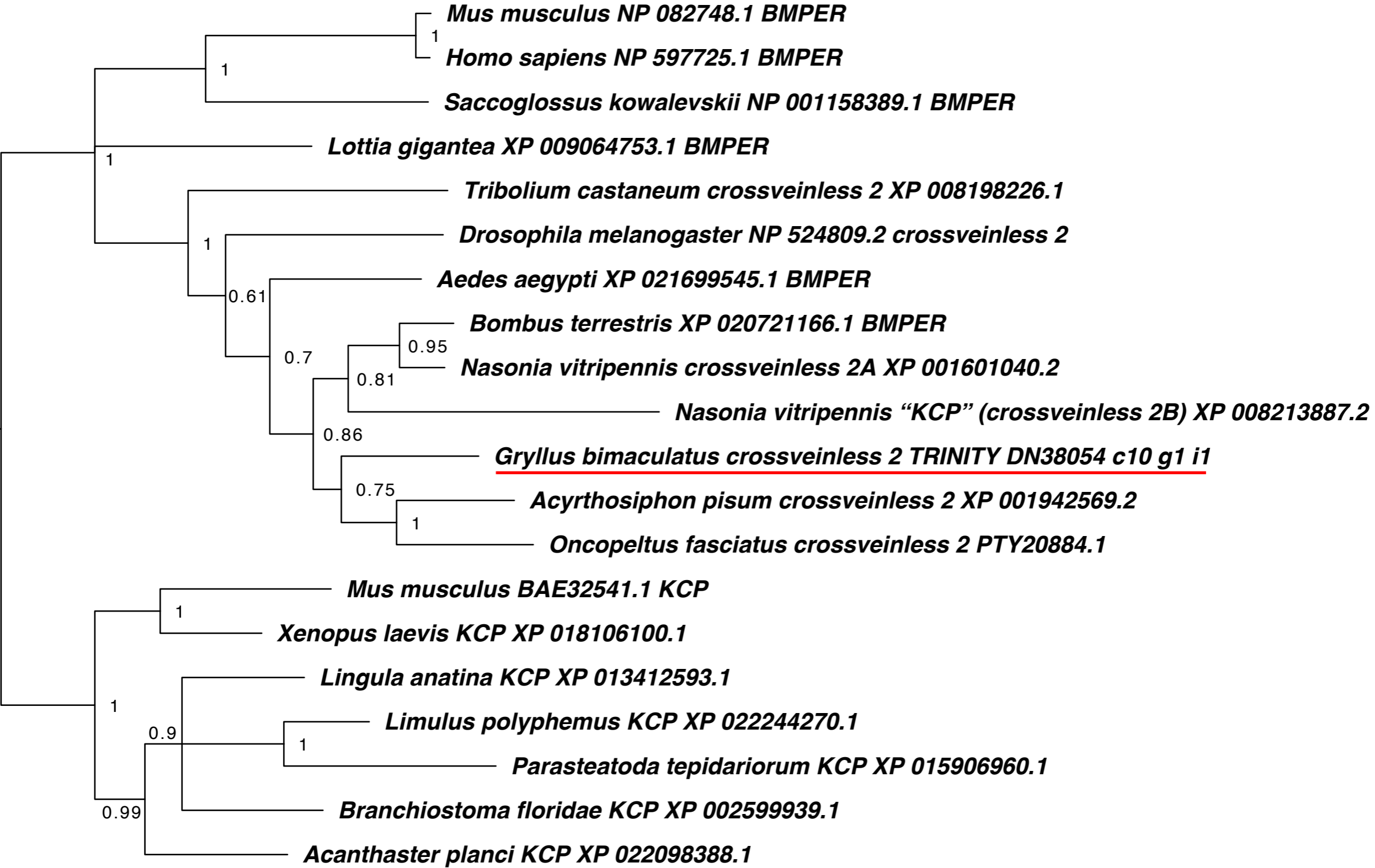

0.2

Supplement: Supplementary file 3. [file elife-68287-supp3.zip › File S3 /TreeFigures/KCP_BMPER_trim.pdf]

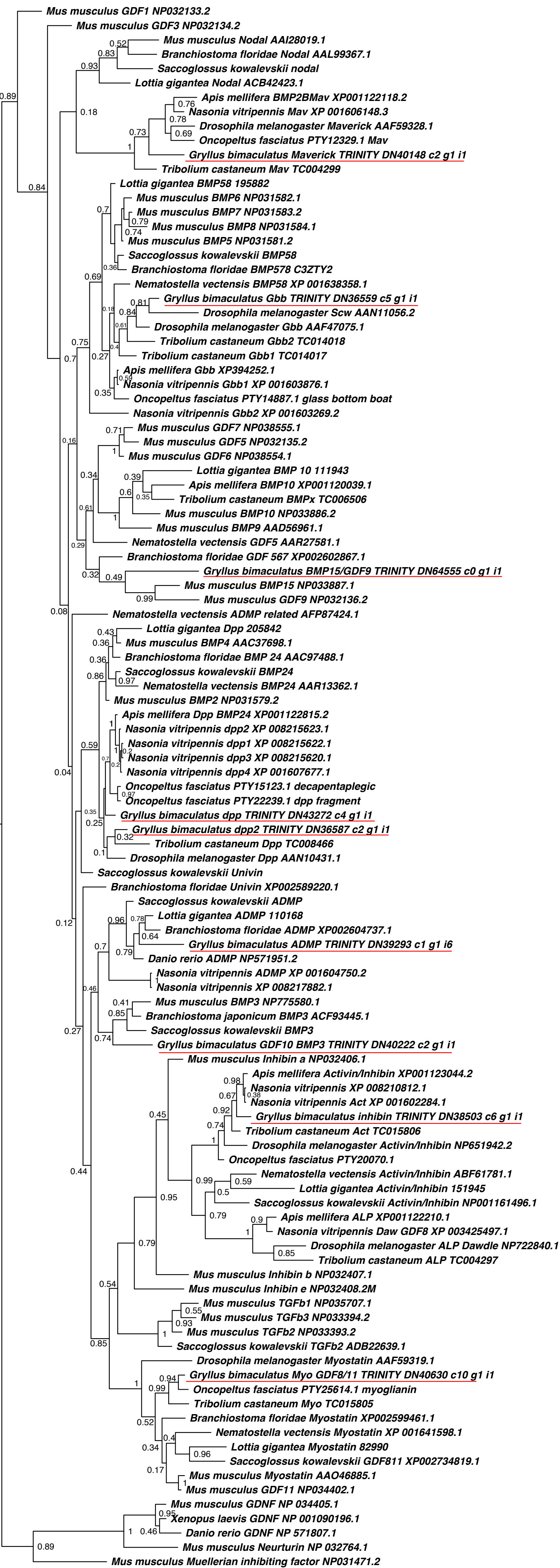

0.4

Supplement: Supplementary file 3. [file elife-68287-supp3.zip › File S3 /TreeFigures/ligands.pdf]

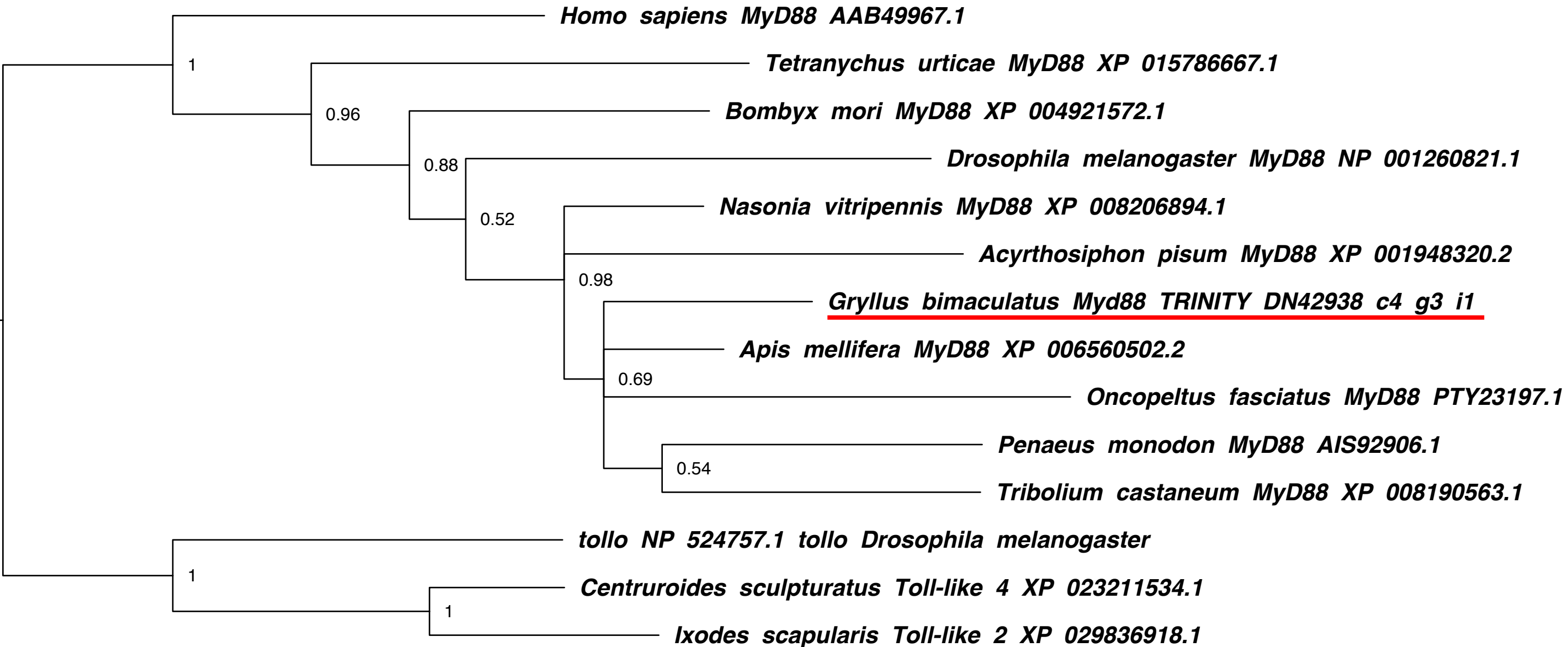

0.2

Supplement: Supplementary file 3. [file elife-68287-supp3.zip › File S3 /TreeFigures/MyD88.pdf]

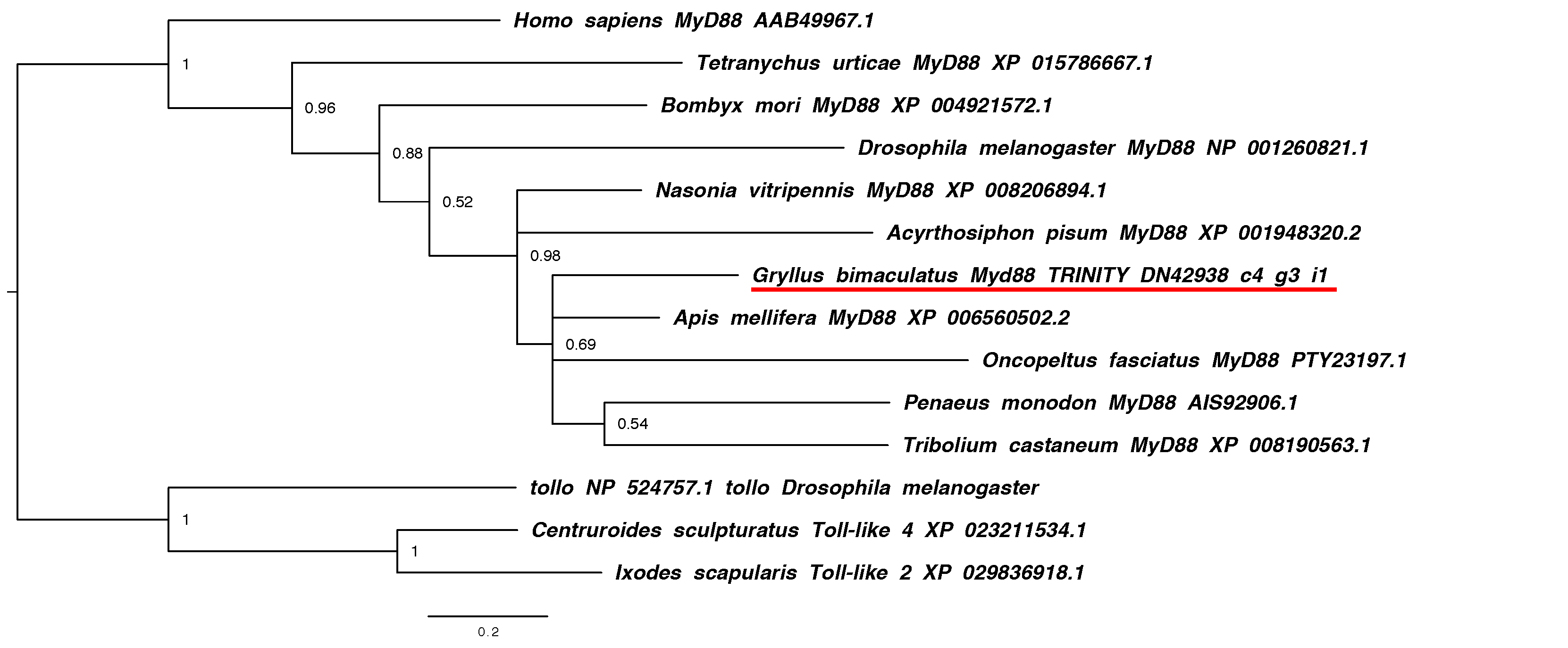

Supplement: Supplementary file 3. [file elife-68287-supp3.zip › File S3 /TreeFigures/MyD88.png]

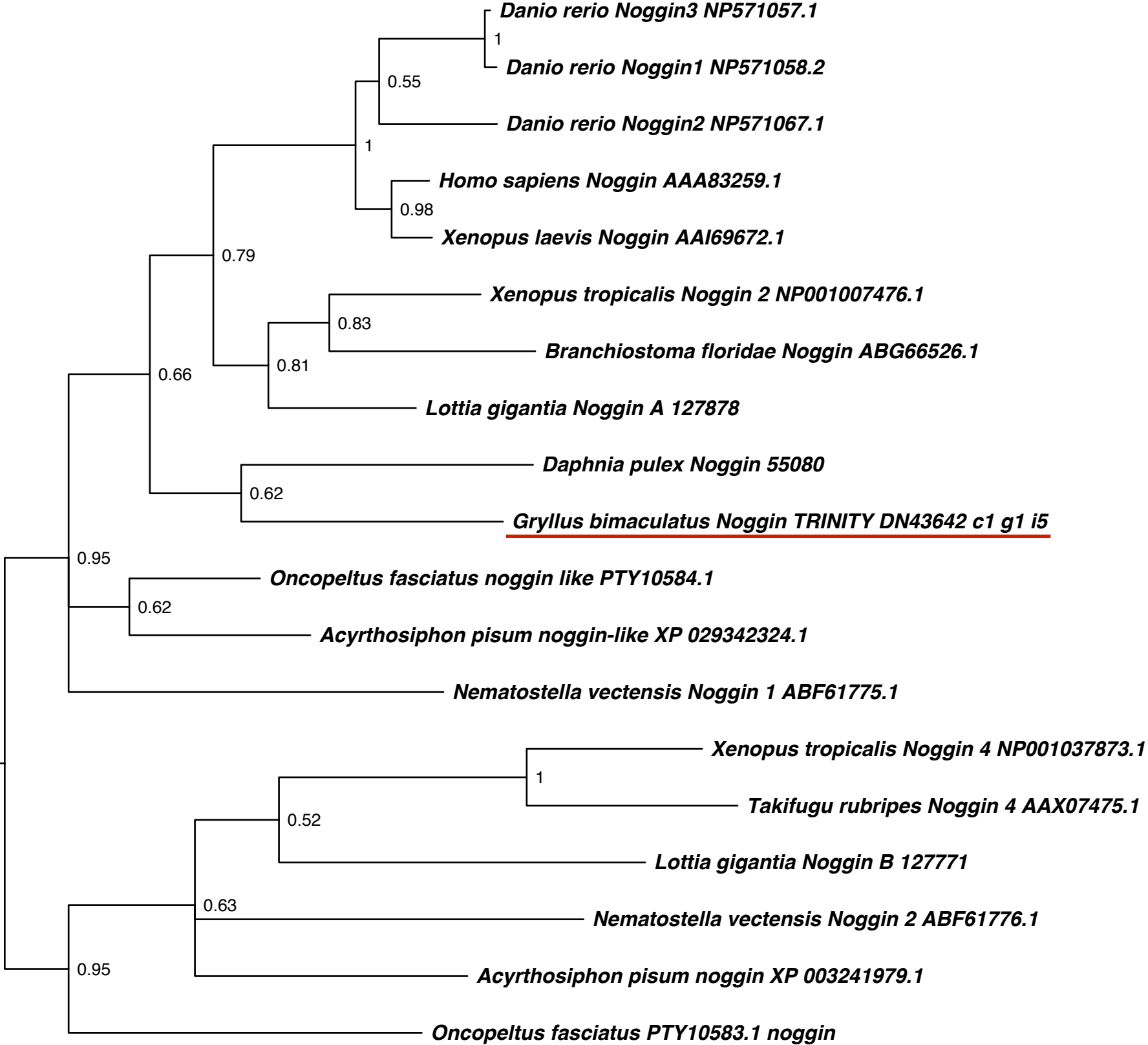

0.2

Supplement: Supplementary file 3. [file elife-68287-supp3.zip › File S3 /TreeFigures/noggin.pdf]

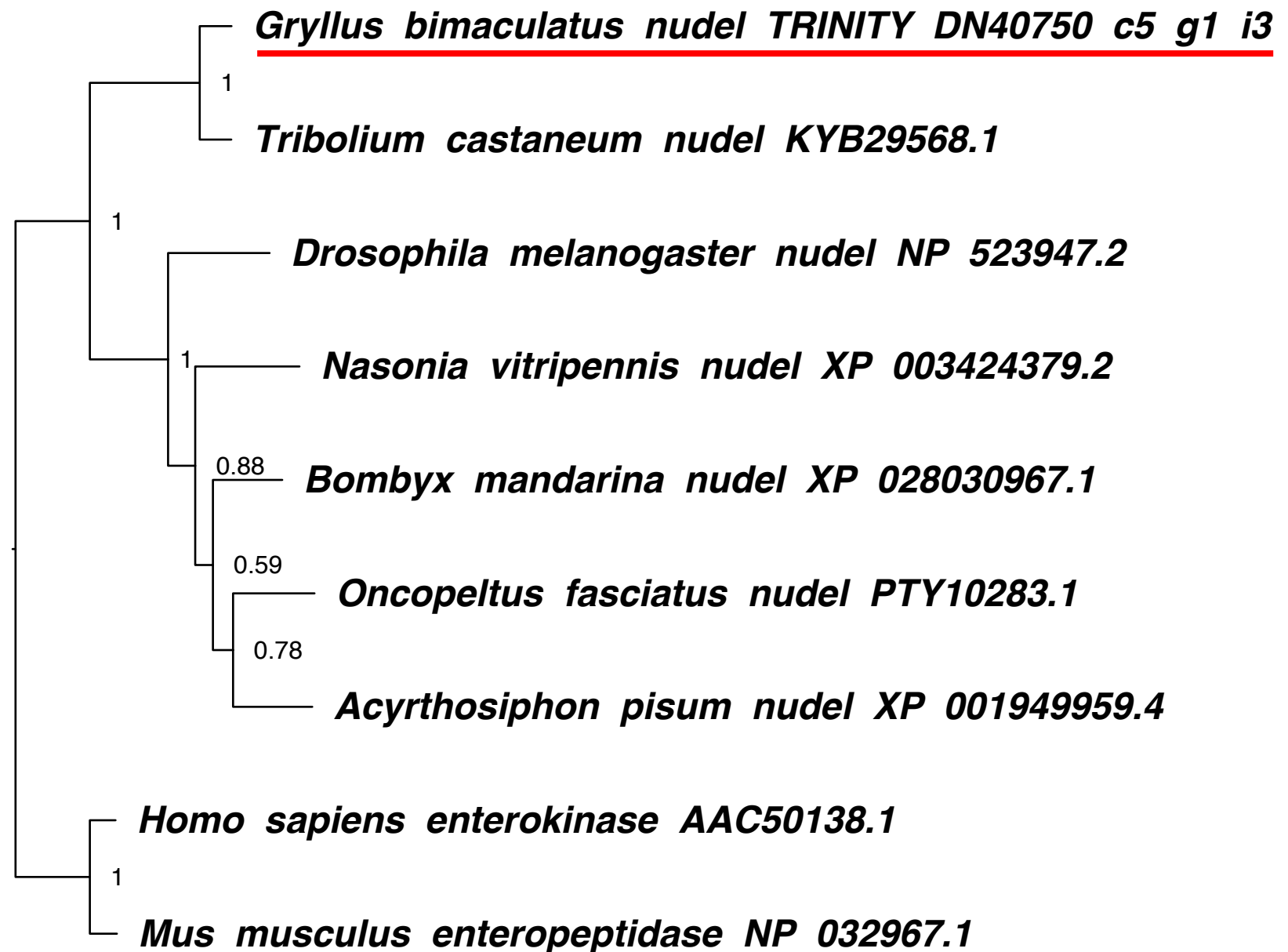

Supplement: Supplementary file 3. [file elife-68287-supp3.zip › File S3 /TreeFigures/nudel.pdf]

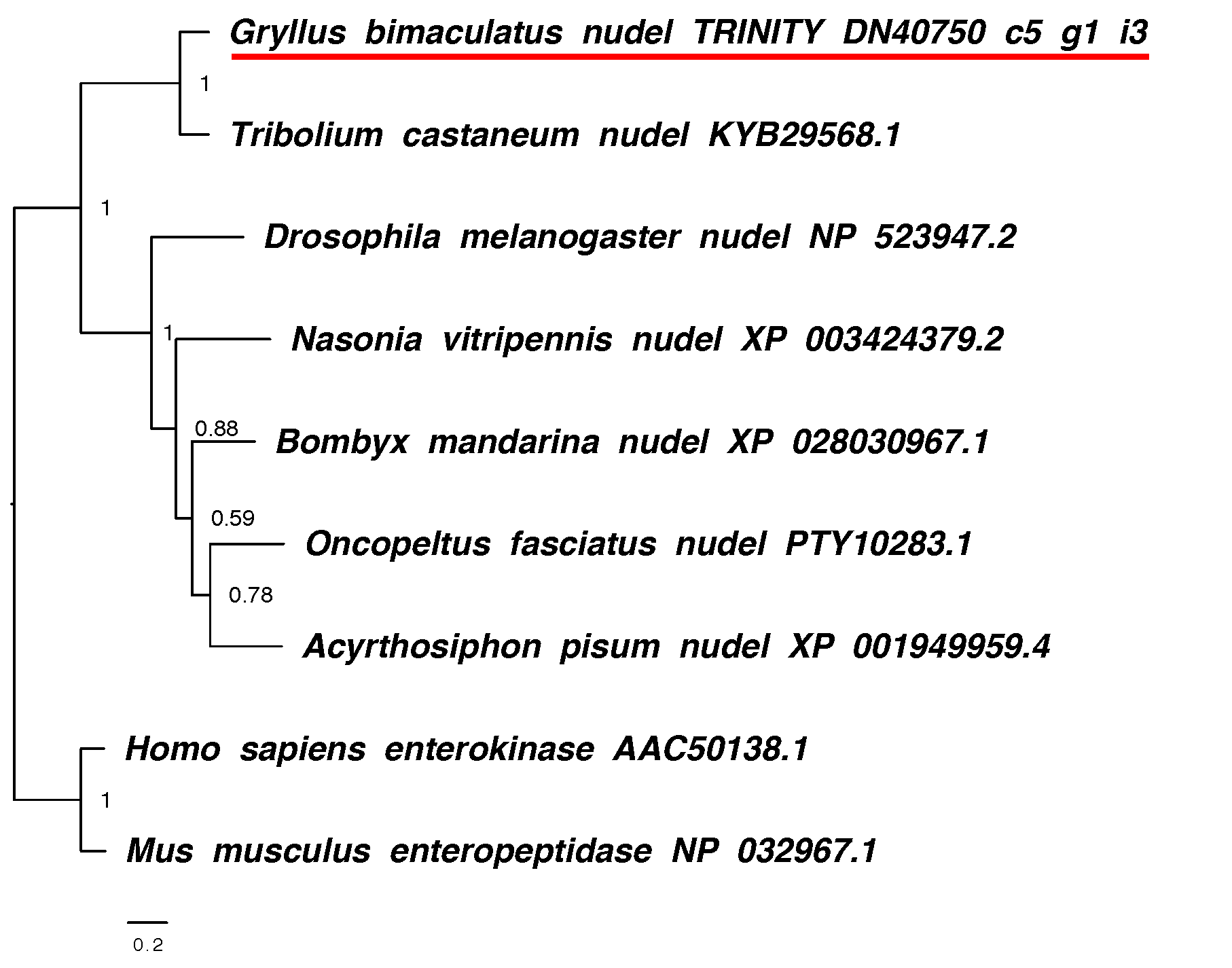

Supplement: Supplementary file 3. [file elife-68287-supp3.zip › File S3 /TreeFigures/nudel.png]

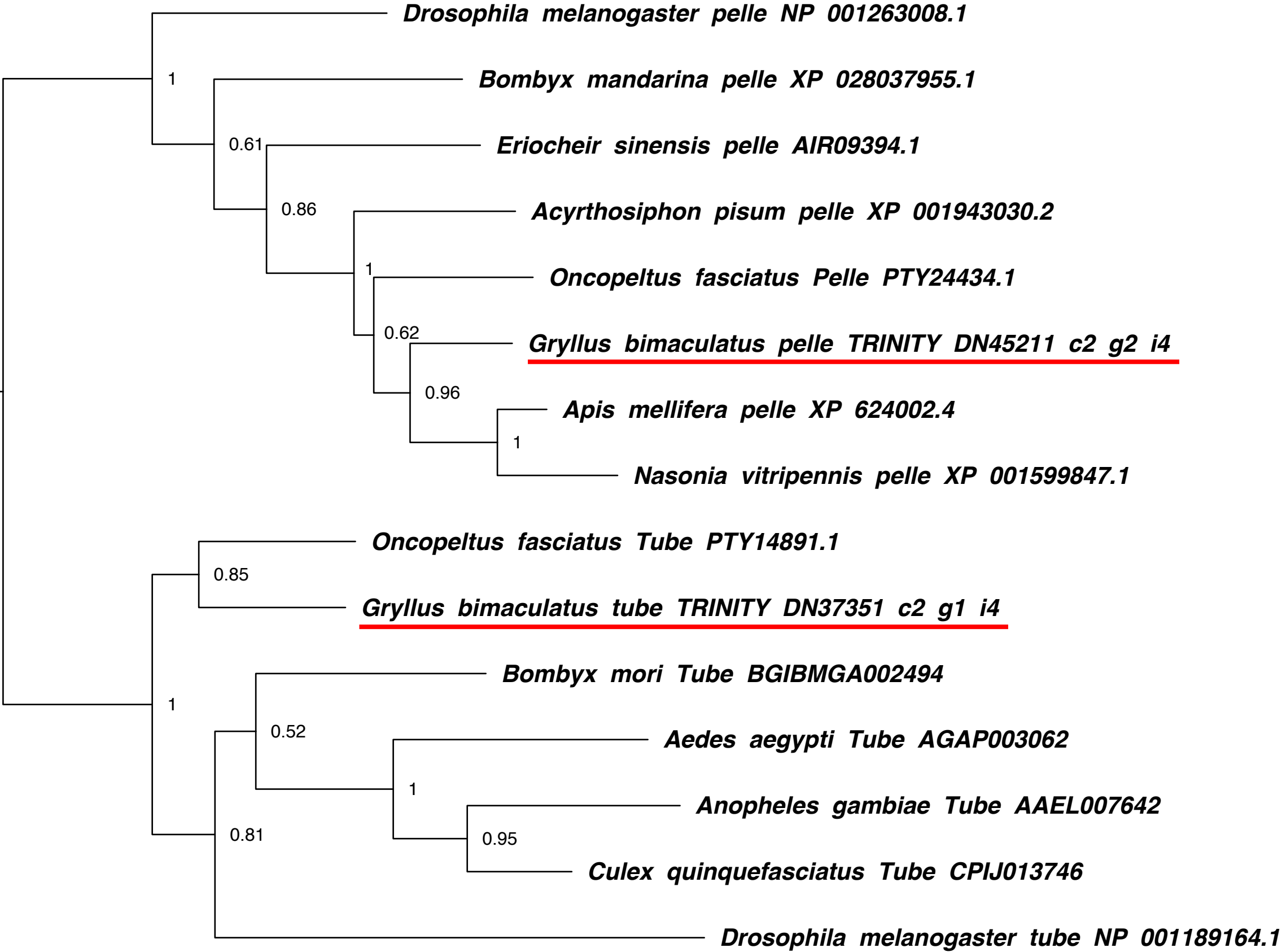

0.3

Supplement: Supplementary file 3. [file elife-68287-supp3.zip › File S3 /TreeFigures/pelle_tube.pdf]

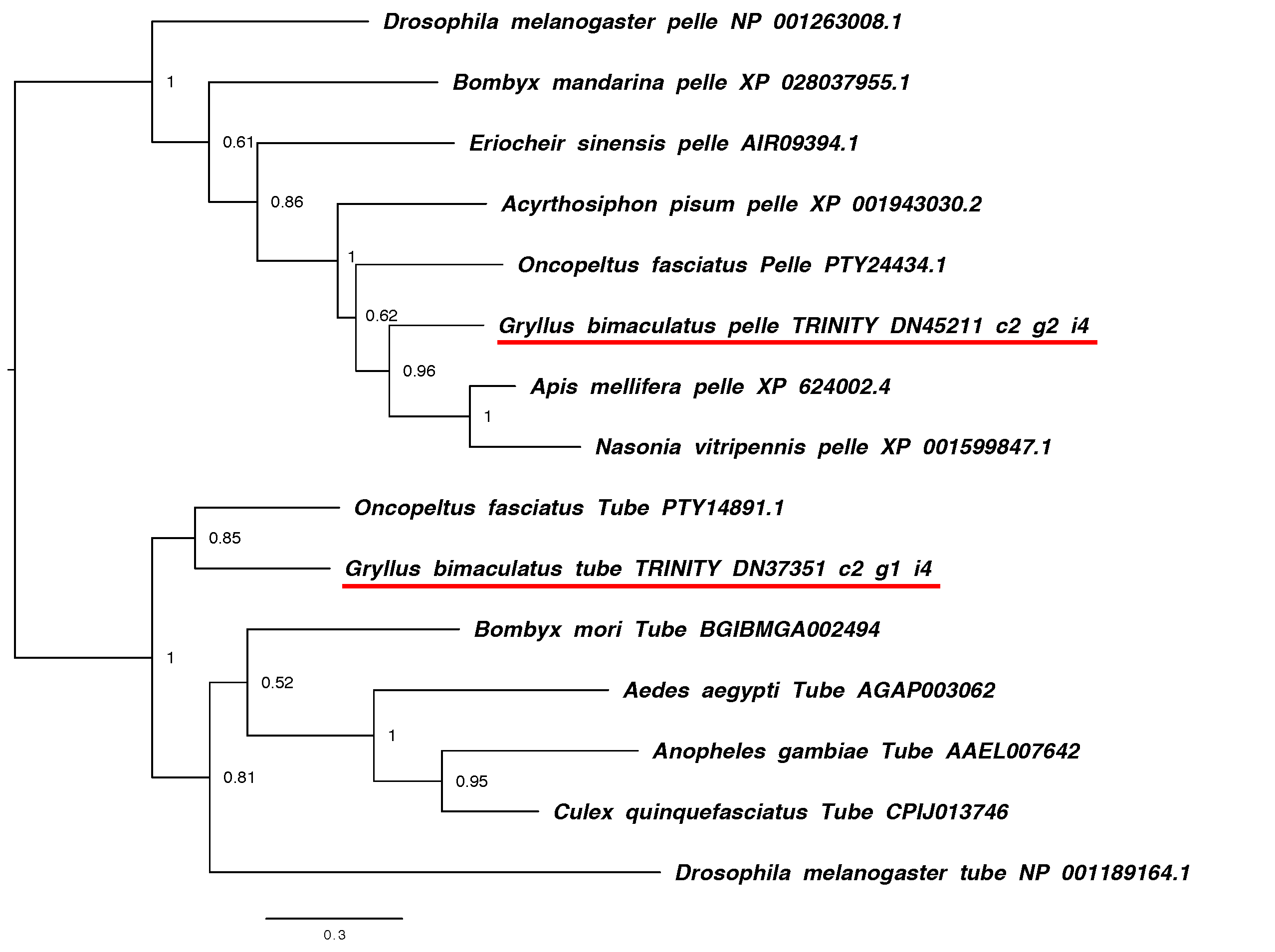

Supplement: Supplementary file 3. [file elife-68287-supp3.zip › File S3 /TreeFigures/pelle_tube.png]

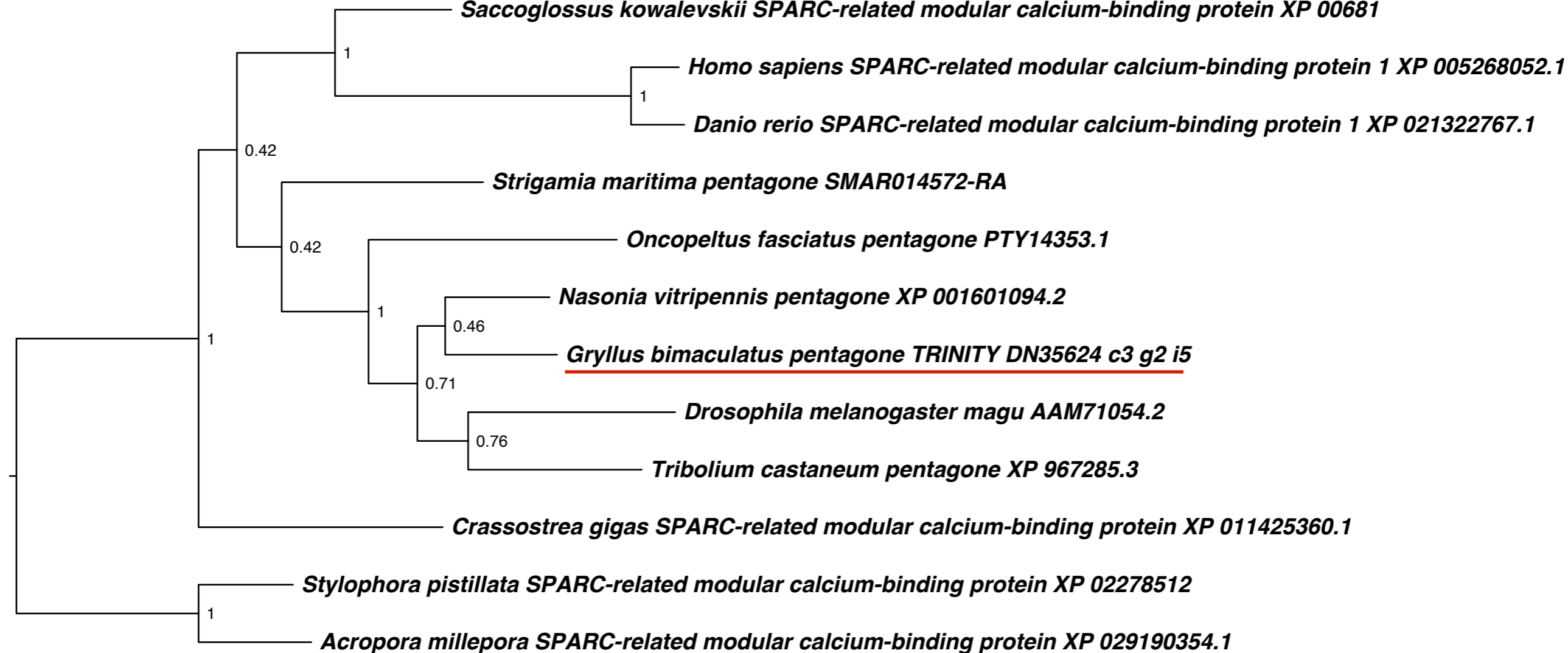

0.2

Supplement: Supplementary file 3. [file elife-68287-supp3.zip › File S3 /TreeFigures/pentagone.pdf]

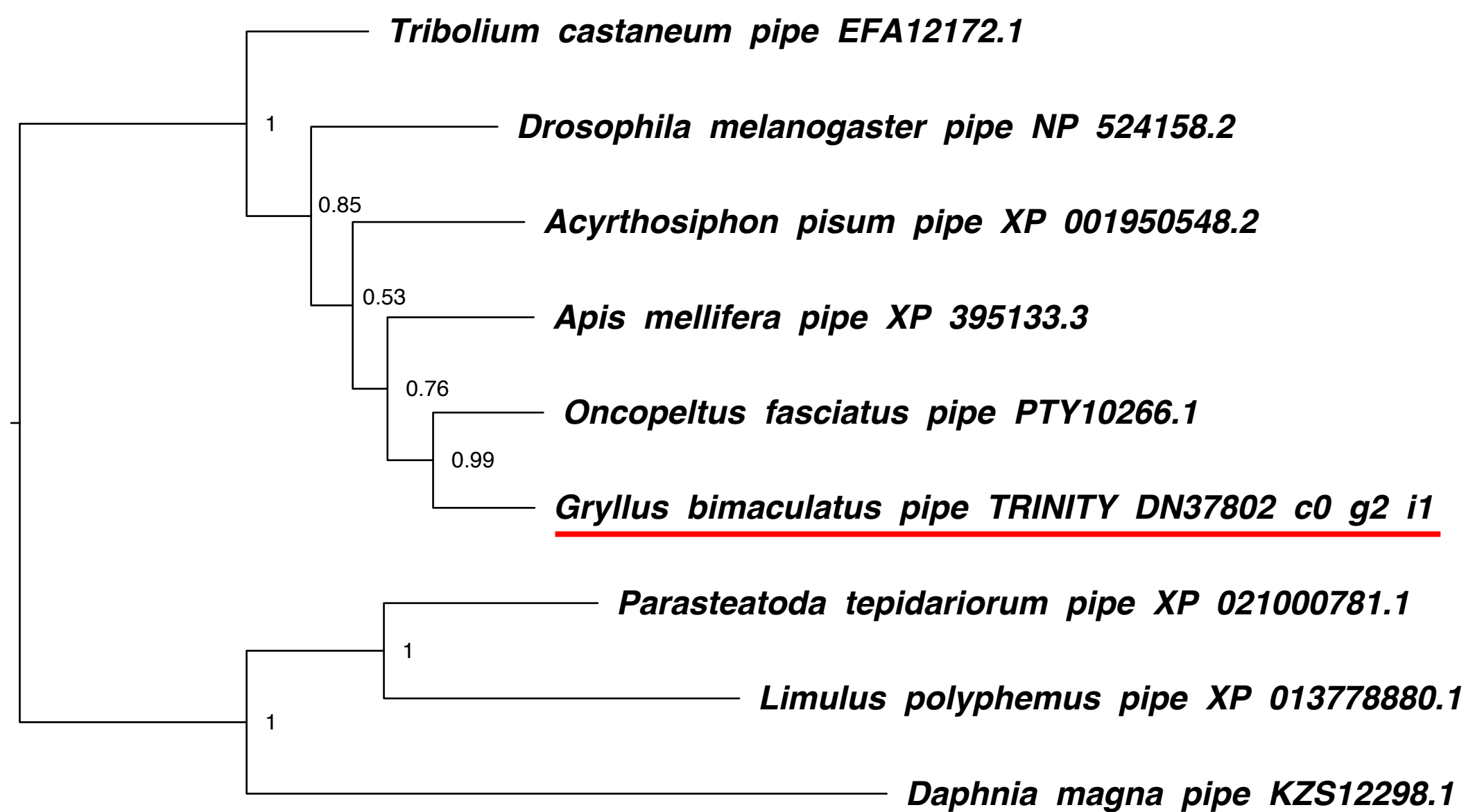

0.2

Supplement: Supplementary file 3. [file elife-68287-supp3.zip › File S3 /TreeFigures/pipe.pdf]

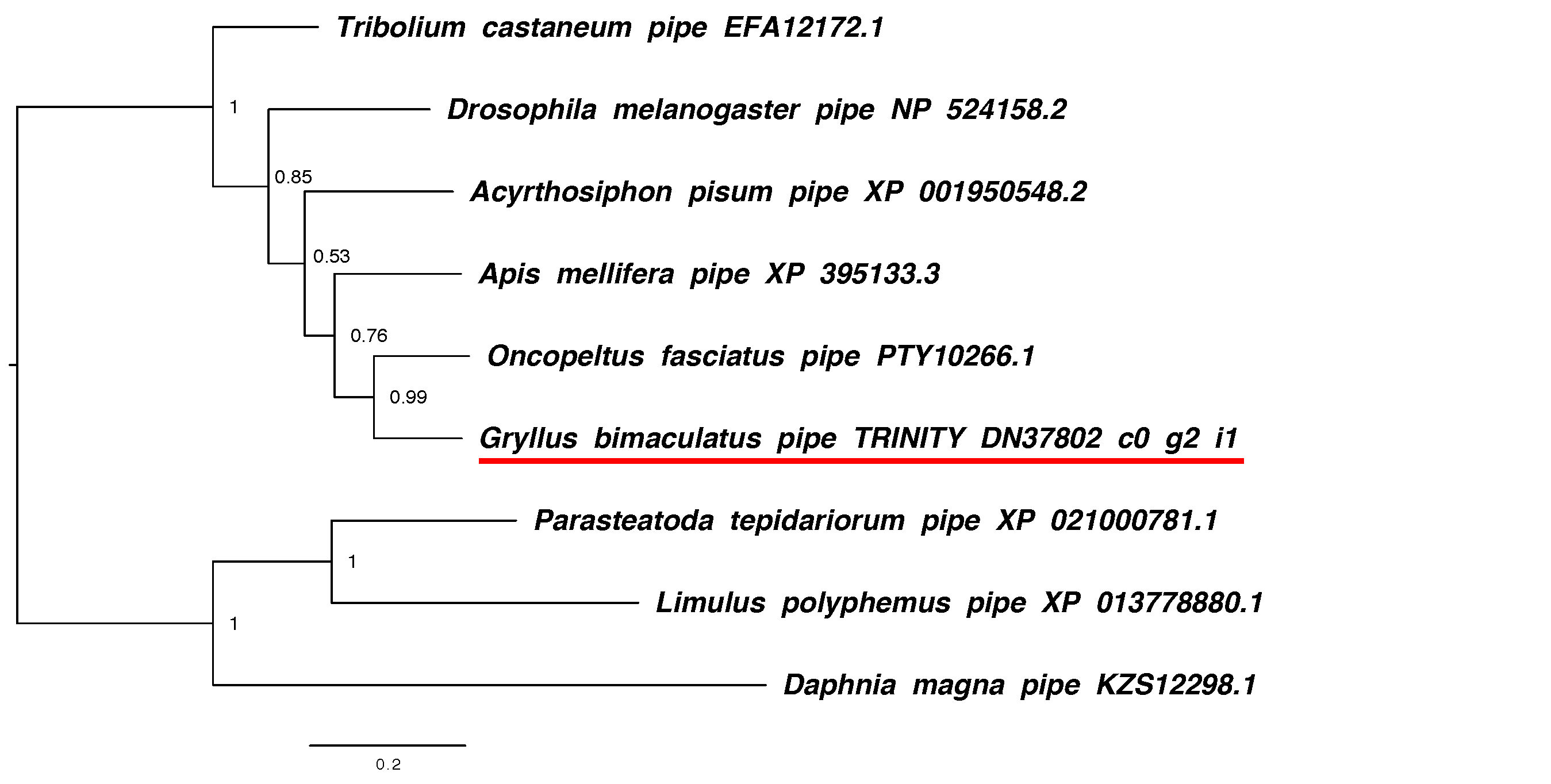

Supplement: Supplementary file 3. [file elife-68287-supp3.zip › File S3 /TreeFigures/pipe.png]

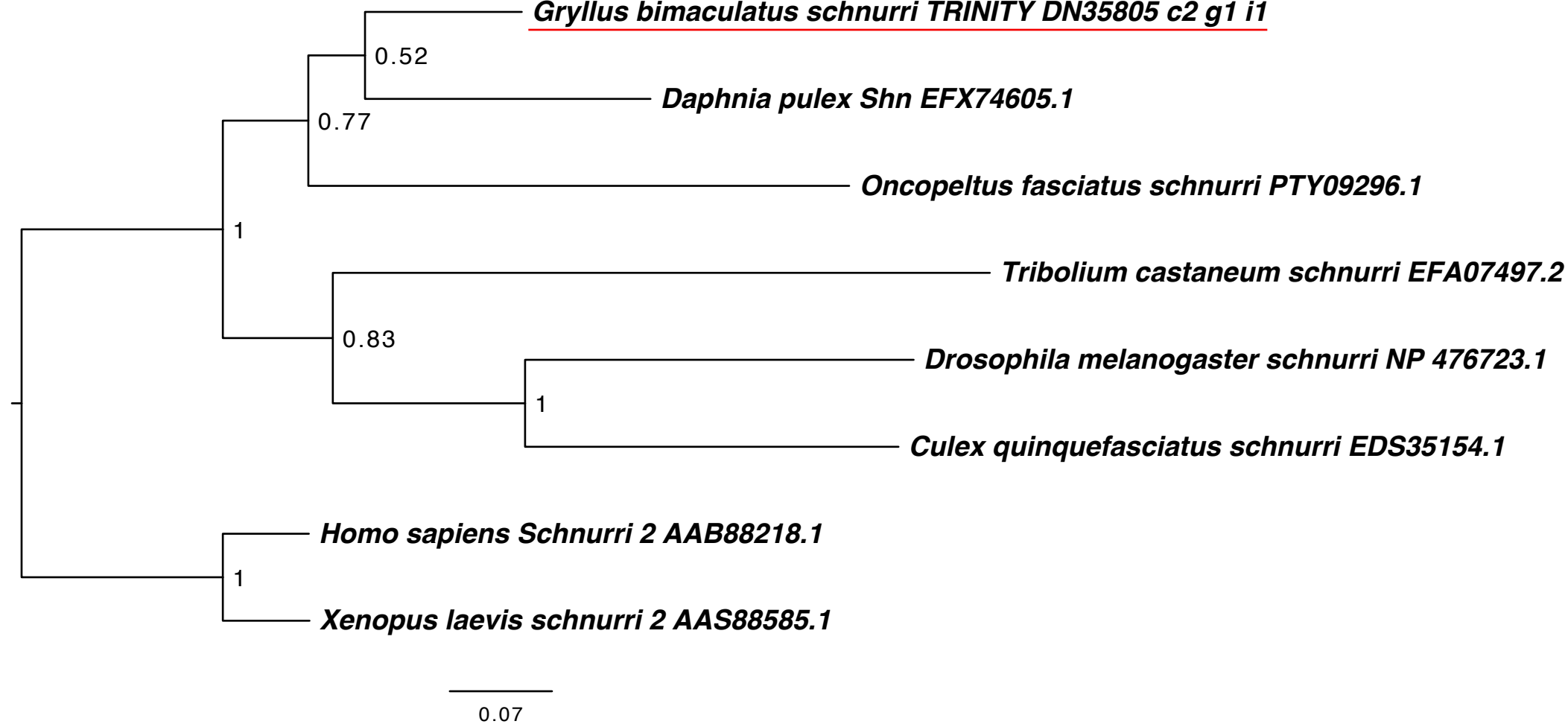

Supplement: Supplementary file 3. [file elife-68287-supp3.zip › File S3 /TreeFigures/schnurri.pdf]

*Tribolium castaneum* MadX TC008788

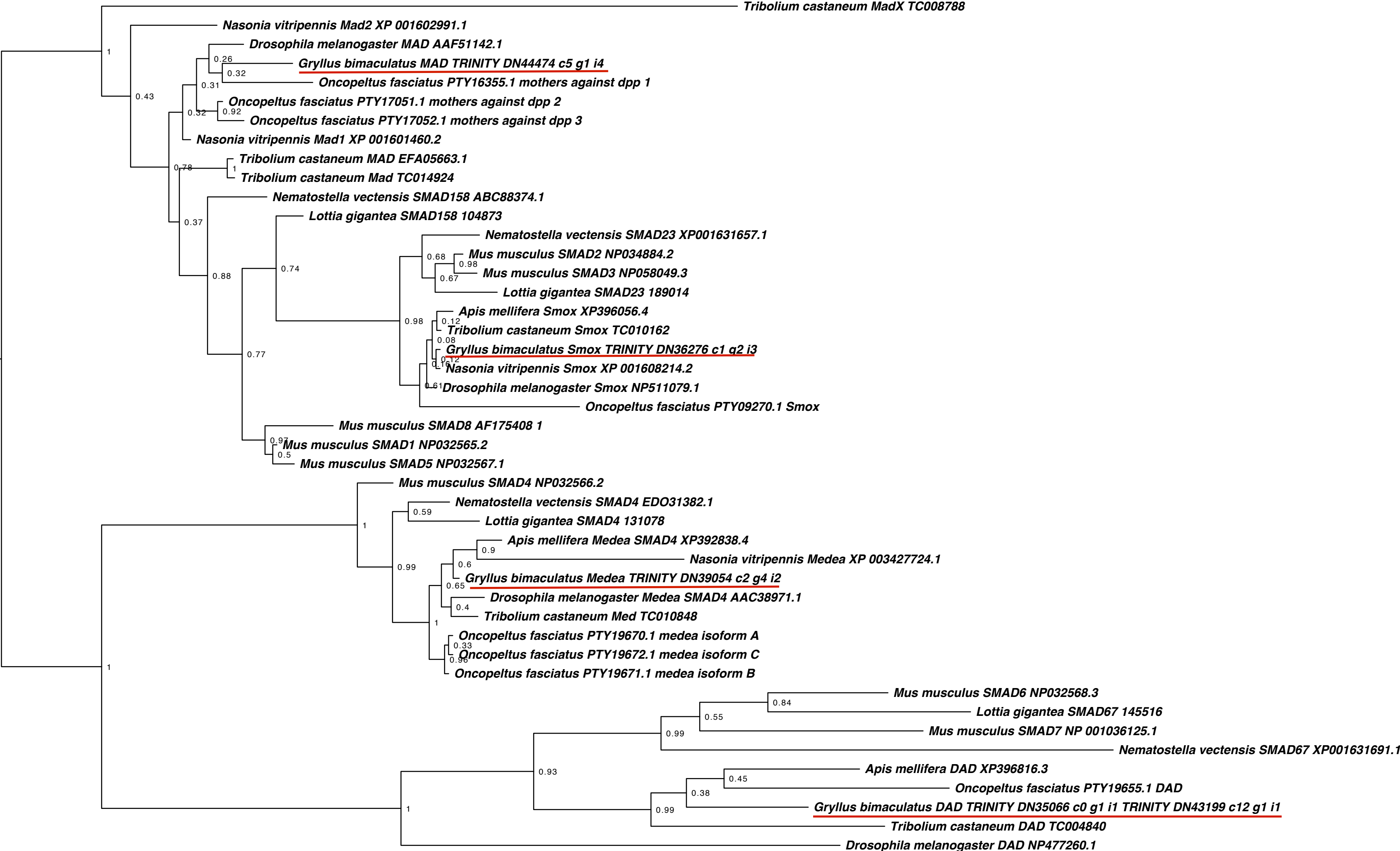

0.2

Supplement: Supplementary file 3. [file elife-68287-supp3.zip › File S3 /TreeFigures/smads.pdf]

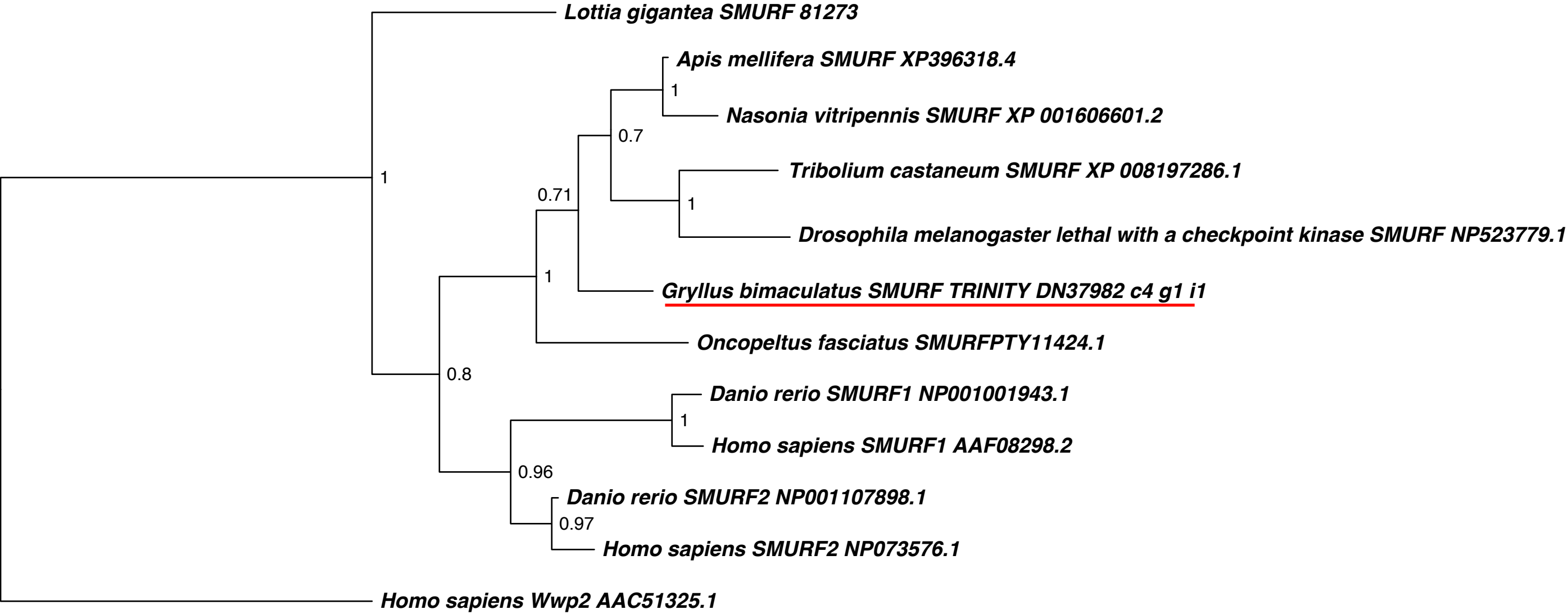

0.08

Supplement: Supplementary file 3. [file elife-68287-supp3.zip › File S3 /TreeFigures/smurf.pdf]

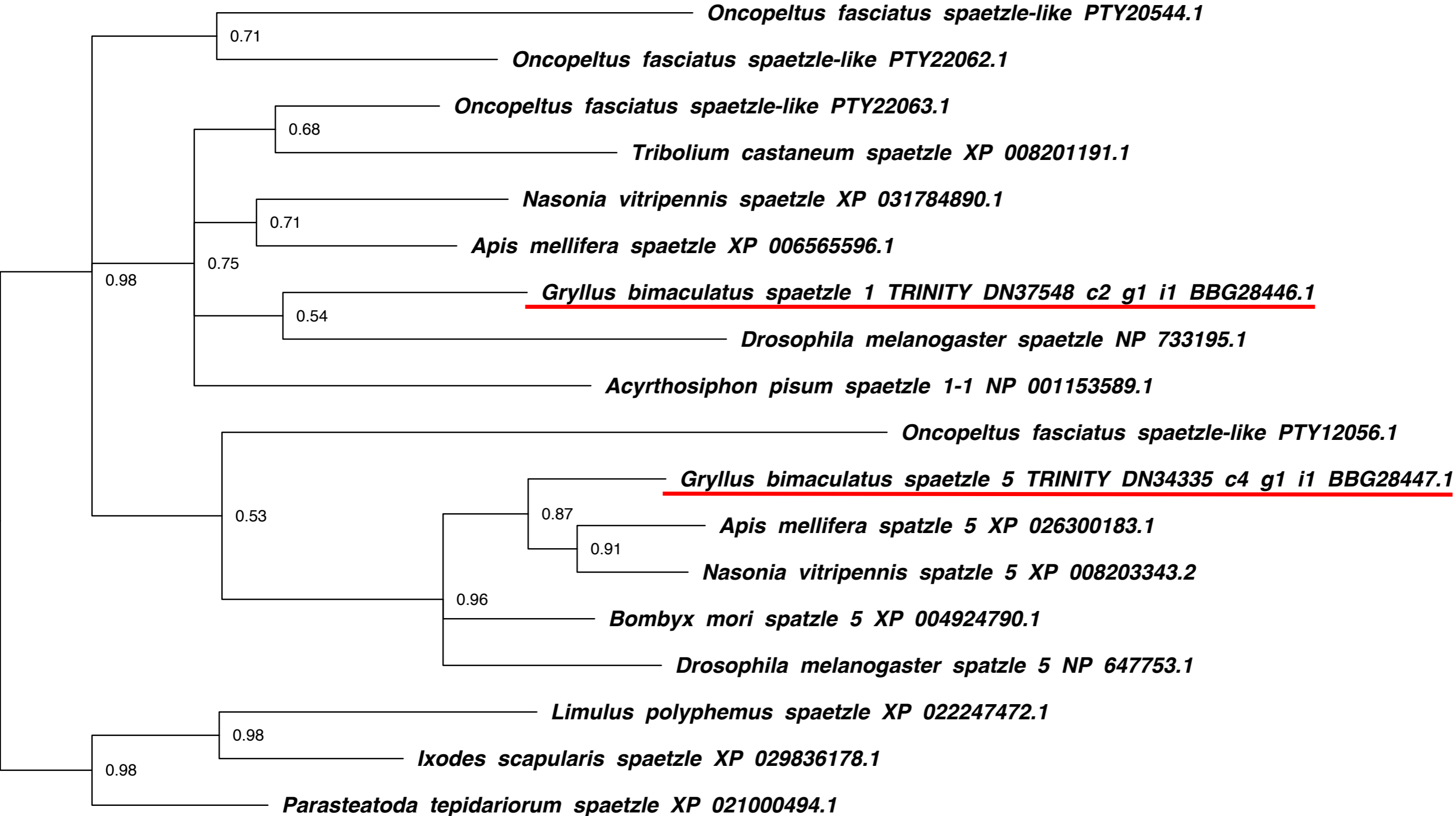

0.2

Supplement: Supplementary file 3. [file elife-68287-supp3.zip › File S3 /TreeFigures/spaetzle.pdf]
