# Supplementary material for: Striking parallels between dorsoventral patterning in Drosophila and Gryllus reveal a complex evolutionary history behind a model gene regulatory network
Source: eLife. 2021 Mar 30;10:e68287. doi: 10.7554/eLife.68287 (PMC8051952; doi:10.7554/eLife.68287)
Supplement: Supplementary file 4. — Where multiple isoforms are present, this is noted in the appropriate column. [file elife-68287-supp4.docx]

| ***TGFβ Ligands*** | |  | *Isoforms?* |  |  | | *Isoforms?* |
| --- | --- | --- | --- | --- | --- | --- | --- |
| ***dpp*** | | *TRINITY_DN43272_c4_g1_i1* |  | ***BAMBI*** | *no clear candidate* | |  |
| ***dpp2*** | | *TRINITY_DN36587_c2_g1_i1* |  | ***Cripto*** | *no clear candidate* | |  |
| ***gbb*** | | *TRINITY_DN36559_c5_g1_i1* |  | ***Crossveinless/Tsg*** | *TRINITY_DN44801_c8_g1_i1* | | *2* |
| ***ADMP*** | | *TRINITY_DN39293_c1_g1_i6* | *5* |  | *TRINITY_DN36254_c5_g1_i3* | | *3* |
| ***BMP3 (GDF10)*** | | *TRINITY_DN40222_c2_g1_i2* | *2* | ***Kielin/Chordin-like*** | *no clear candidate* | |  |
| ***Myostatin (D, T)*** | | *TRINITY_DN40630_c10_g1_i1* |  | ***Crossveinless 2/ BMPER*** | *TRINITY_DN38054_c10_g1_i1* | |  |
| ***Inhibin/Activin*** | *TRINITY_DN38503_c6_g1_i1* | | *2* | ***Dan*** | *TRINITY_DN43618_c3_g1_i1* |  | |
| ***Maverick*** | *TRINITY_DN40148_c2_g1_i1* | |  |  |  |  | |
| ***BMP15/GDF9*** | *TRINITY_DN64555_c0_g1_i1* | |  | ***Gremlin*** | *TRINITY_DN44207_c6_g1_i1* |  | |
| ***ALKs*** |  | |  |  |  |  | |
| ***BMP receptor1*** | *TRINITY_DN40849_c4_g1_i4* | | *4* | ***Follistatin*** | *TRINITY_DN42584_c3_g1_i1* |  | |
| ***TGF receptor 1 (?)*** | *TRINITY_DN34065_c3_g1_i3* | | *2* |  |  |  | |
| ***activin receptor saxophone*** | *TRINITY_DN39845_c0_g1_i1* | |  | ***Noggin*** | *TRINITY_DN43642_c1_g1* | *2* | |
| ***activin receptor 2*** | *TRINITY_DN35393_c5_g2_i2* | | *2* |  |  |  | |
| ***BMP receptor 2*** | *TRINITY_DN39452_c4_g1_i1* | | *2* | ***Short gastrulation /Chordin*** | *no clear candidate* |  | |
| ***SMADs*** |  | |  |  |  |  | |
| ***MAD*** | *TRINITY_DN44474_c5_g1* | | *4* | ***SMURF*** | *TRINITY_DN37982_c4_g1_i1* |  | |
| ***DAD*** | *TRINITY_DN43199_c12_g1_i1* | |  |  |  |  | |
| ***Medea*** | *TRINITY_DN39054_c2_g4* | | *2* | ***Tolloid*** | *TRINITY_DN35005_c2_g1* | *2* | |
| ***Smox*** | *TRINITY_DN36276_c1_g2_i7* | | *3* |  |  |  | |
|  |  | |  | ***Pentagone:*** | *TRINITY_DN35624_c3_g2_i5* | *10* | |
| ***Brinker*** | *TRINITY_DN38911_c1_g1_i1* | |  |  |  |  | |
|  |  | |  | ***Schnurri:*** | *TRINITY_DN35805_c2_g1_i1* |  | |

Supplementary file 4*.* Recovery of the TGFβ/BMP pathway of *G. bimaculatus,* Summary. Where multiple isoforms are present, this is noted in the appropriate column.
