# Supplementary material for: Striking parallels between dorsoventral patterning in Drosophila and Gryllus reveal a complex evolutionary history behind a model gene regulatory network
Source: eLife. 2021 Mar 30;10:e68287. doi: 10.7554/eLife.68287 (PMC8051952; doi:10.7554/eLife.68287)
Supplement: Supplementary file 5. [file elife-68287-supp5.docx]

|  | |  |
| --- | --- | --- |
| ***pipe*** | | *TRINITY_DN37802_c0_g2_i1* |
| ***windbeutel*** | | *TRINITY_DN37576_c7_g1_i2* |
| ***nudel*** | | *TRINITY_DN40750_c5_g1_i3* |
| ***spätzle 1-like*** | | *TRINITY_DN37548_c2_g1_i1* |
| ***spätzle 5-like*** | | *TRINITY_DN34335_c4_g1_i1* |
| ***Toll1*** | | *TRINITY_DN36150_c1_g2_i4* |
| ***Myd88*** | *TRINITY_DN42938_c4_g3_i1* | |
| ***tube*** | *TRINITY_DN37351_c2_g1_i4* | |
| ***pelle*** | *TRINITY_DN45211_c2_g2_i4* | |
| ***cactus*** | *TRINITY_DN40207_c4_g2_i2* | |
| ***dorsal 1*** | *TRINITY_DN38047_c8_g1_i5* | |
| ***dorsal 2*** | *TRINITY_DN41035_c5_g1_i1* | |
| ***dorsal 3*** | *TRINITY_DN44709_c6_g1_i1* | |

Supplementary file 5. Recovery of Toll pathway components of *G. bimaculatus*
