# Supplementary material for: Striking parallels between dorsoventral patterning in Drosophila and Gryllus reveal a complex evolutionary history behind a model gene regulatory network
Source: eLife. 2021 Mar 30;10:e68287. doi: 10.7554/eLife.68287 (PMC8051952; doi:10.7554/eLife.68287)
Supplement: Transparent reporting form [file elife-68287-transrepform.pdf]

Westbrook Centre
Milton Road
Cambridge CB4 1YG, UK
P 01223 855340
W elifesciences.org
T @elife

eLife's transparent reporting form

We encourage authors to provide detailed information *within their submission* to facilitate the interpretation and replication of experiments. Authors can upload supporting documentation to indicate the use of appropriate reporting guidelines for health-related research (see EQUATOR Network), life science research (see the BioSharing Information Resource), or the ARRIVE guidelines for reporting work involving animal research. Where applicable, authors should refer to any relevant reporting standards documents in this form.

If you have any questions, please consult our Journal Policies and/or contact us: editorial@elifesciences.org.

Sample-size estimation

- You should state whether an appropriate sample size was computed when the study was being designed
- You should state the statistical method of sample size computation and any required assumptions
- If no explicit power analysis was used, you should describe how you decided what sample (replicate) size (number) to use

Please outline where this information can be found within the submission (e.g., sections or figure legends), or explain why this information doesn't apply to your submission:

> No ethical considerations regarding sample size were necessary as the study deals with common laboratory insect model (Gryllus). The validation of experimental results did not require a prior calculation of sample-size since the described knockdown experiments resulted in highly penetrant phenotypes.

Replicates

- You should report how often each experiment was performed
- You should include a definition of biological versus technical replication
- The data obtained should be provided and sufficient information should be provided to indicate the number of independent biological and/or technical replicates
- If you encountered any outliers, you should describe how these were handled
- Criteria for exclusion/inclusion of data should be clearly stated
- High-throughput sequence data should be uploaded before submission, with a private link for reviewers provided (these are available from both GEO and ArrayExpress)

Please outline where this information can be found within the submission (e.g., sections or figure legends), or explain why this information doesn't apply to your submission:

> The conclusions of the paper rest on RNAi knockdown experiments which were performed according to the highest standards of the field (as in our former studies). The experimental procedure is reported in the section "Parental RNAi experiments" (see Material and Methods). RNAi knockdown for Gryllus Toll1 was repeated using nonoverlapping fragments to test for variation in the knockdown strength and potential second site effects. There was no indication for second site effects. Furthermore, the independent knockdown of components of the same pathway produced an identical or nearly identical spectrum of phenotypes. This provides mutual support for the specificity of the knockdown phenotypes.

eLife Sciences Publications, Ltd is a limited liability non-profit non-stock corporation incorporated in the State of Delaware, USA, with company number 5030732, and is registered in the UK with company number FC030576 and branch number BR015634 at the address Westbrook Centre, Milton Road, Cambridge CB4 1YG.

Statistical reporting

- Statistical analysis methods should be described and justified
- Raw data should be presented in figures whenever informative to do so (typically when N per group is less than 10)
- For each experiment, you should identify the statistical tests used, exact values of N, definitions of center, methods of multiple test correction, and dispersion and precision measures (e.g., mean, median, SD, SEM, confidence intervals; and, for the major substantive results, a measure of effect size (e.g., Pearson's r, Cohen's d)
- Report exact p-values wherever possible alongside the summary statistics and 95% confidence intervals. These should be reported for all key questions and not only when the p-value is less than 0.05.

Please outline where this information can be found within the submission (e.g., sections or figure legends), or explain why this information doesn't apply to your submission:

> Programmes and statistical procedures used for the generation and annotation of the transcriptome, for the phylogeny reconstruction and the HHM-based identification of sog fragments are described in "Materials and Methods".
> Numbers of injected embryos and classification of phenotypes can be found in Figure 3 - figure supplement 1.

(For large datasets, or papers with a very large number of statistical tests, you may upload a single table file with tests, Ns, etc., with reference to sections in the manuscript.)

Group allocation

- Indicate how samples were allocated into experimental groups (in the case of clinical studies, please specify allocation to treatment method); if randomization was used, please also state if restricted randomization was applied
- Indicate if masking was used during group allocation, data collection and/or data analysis

Please outline where this information can be found within the submission (e.g., sections or figure legends), or explain why this information doesn't apply to your submission:

> Not applicable.

Additional data files ("source data")

- We encourage you to upload relevant additional data files, such as numerical data that are represented as a graph in a figure, or as a summary table
- Where provided, these should be in the most useful format, and they can be uploaded as "Source data" files linked to a main figure or table
- Include model definition files including the full list of parameters used
- Include code used for data analysis (e.g., R, MatLab)
- Avoid stating that data files are "available upon request"

Please indicate the figures or tables for which source data files have been provided:

> Supplementary file 1 contains the data from HMM search for short gastrulation homologs in Orthoptera.
> Supplementary file 2 contains the results from transcriptome annotation based on an automated Blast approach.
> Supplementary file 3 contains sequences of genes coding for BMP and Toll pathway components and their alignments used for phylogenetic reconstruction.

eLife Sciences Publications, Ltd is a limited liability non-profit non-stock corporation incorporated in the State of Delaware, USA, with company number 5030732, and is registered in the UK with company number FC030576 and branch number BR015634 at the address Westbrook Centre, Milton Road, Cambridge CB4 1YG.
