# Supplementary material for: Striking parallels between dorsoventral patterning in Drosophila and Gryllus reveal a complex evolutionary history behind a model gene regulatory network
Source: eLife. 2021 Mar 30;10:e68287. doi: 10.7554/eLife.68287 (PMC8051952; doi:10.7554/eLife.68287)
Supplement: Supplementary file 3. [file elife-68287-supp3.zip › File S3 /TreeFigures/tolloid.pdf]

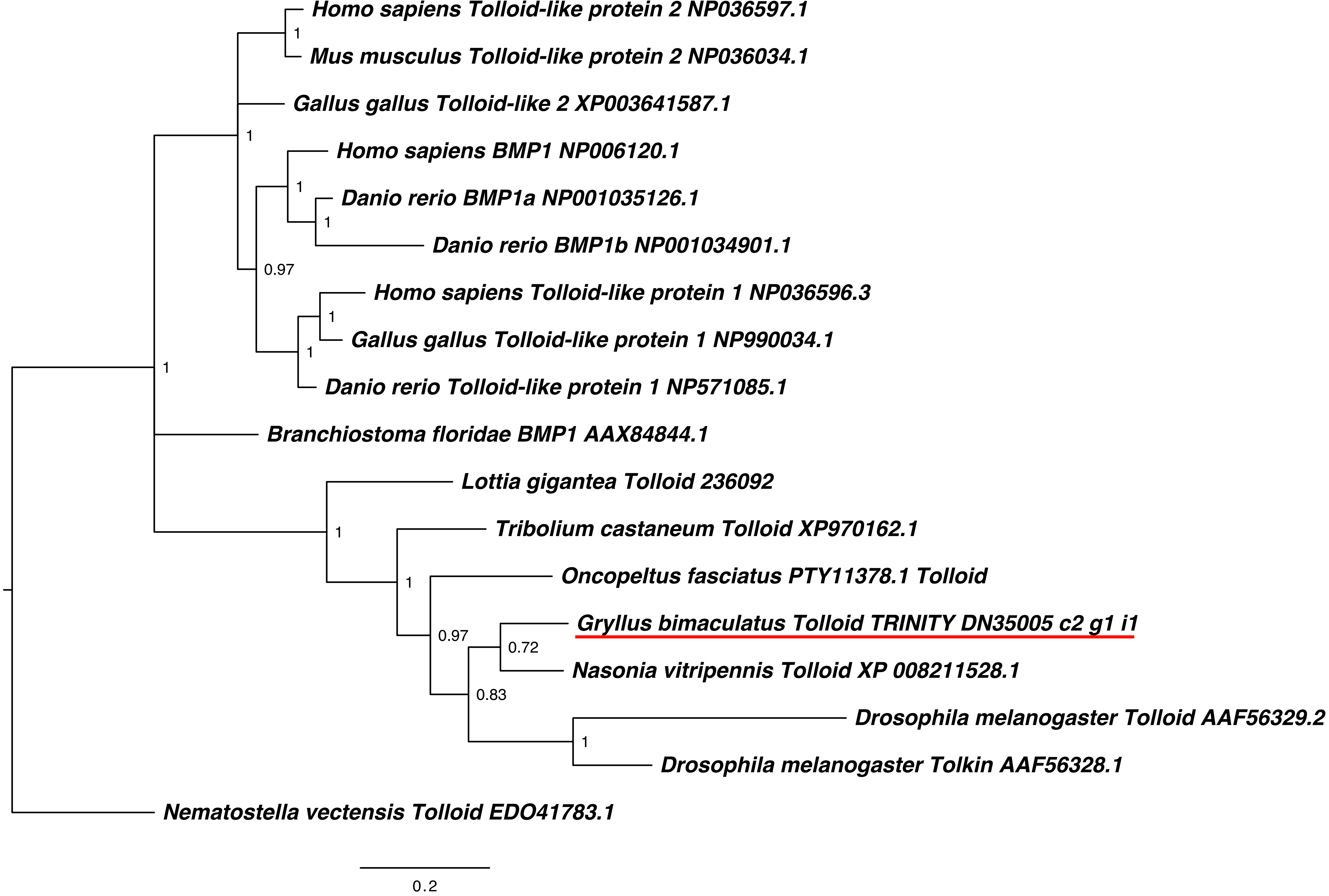
Homo sapiens Tolloid-like protein 2 NP036597.1
Mus musculus Tolloid-like protein 2 NP036034.1
Gallus gallus Tolloid-like 2 XP003641587.1
Homo sapiens BMP1 NP006120.1
Danio rerio BMP1a NP001035126.1
Danio rerio BMP1b NP001034901.1
Homo sapiens Tolloid-like protein 1 NP036596.3
Gallus gallus Tolloid-like protein 1 NP990034.1
Danio rerio Tolloid-like protein 1 NP571085.1
Branchiostoma floridae BMP1 AAX84844.1
Lottia gigantea Tolloid 236092
Tribolium castaneum Tolloid XP970162.1
Oncopeltus fasciatus PTY11378.1 Tolloid
Gryllus bimaculatus Tolloid TRINITY DN35005 c2 g1 i1
Nasonia vitripennis Tolloid XP 008211528.1
Drosophila melanogaster Tolloid AAF56329.2
Drosophila melanogaster Tolkin AAF56328.1
Nematostella vectensis Tolloid EDO41783.1
1
1
1
1
1
0.97
1
1
1
1
1
0.97
0.72
0.83
1
0.2
