# Supplementary material for: Striking parallels between dorsoventral patterning in Drosophila and Gryllus reveal a complex evolutionary history behind a model gene regulatory network
Source: eLife. 2021 Mar 30;10:e68287. doi: 10.7554/eLife.68287 (PMC8051952; doi:10.7554/eLife.68287)
Supplement: Supplementary file 3. [file elife-68287-supp3.zip › File S3 /TreeFigures/Tsg.pdf]

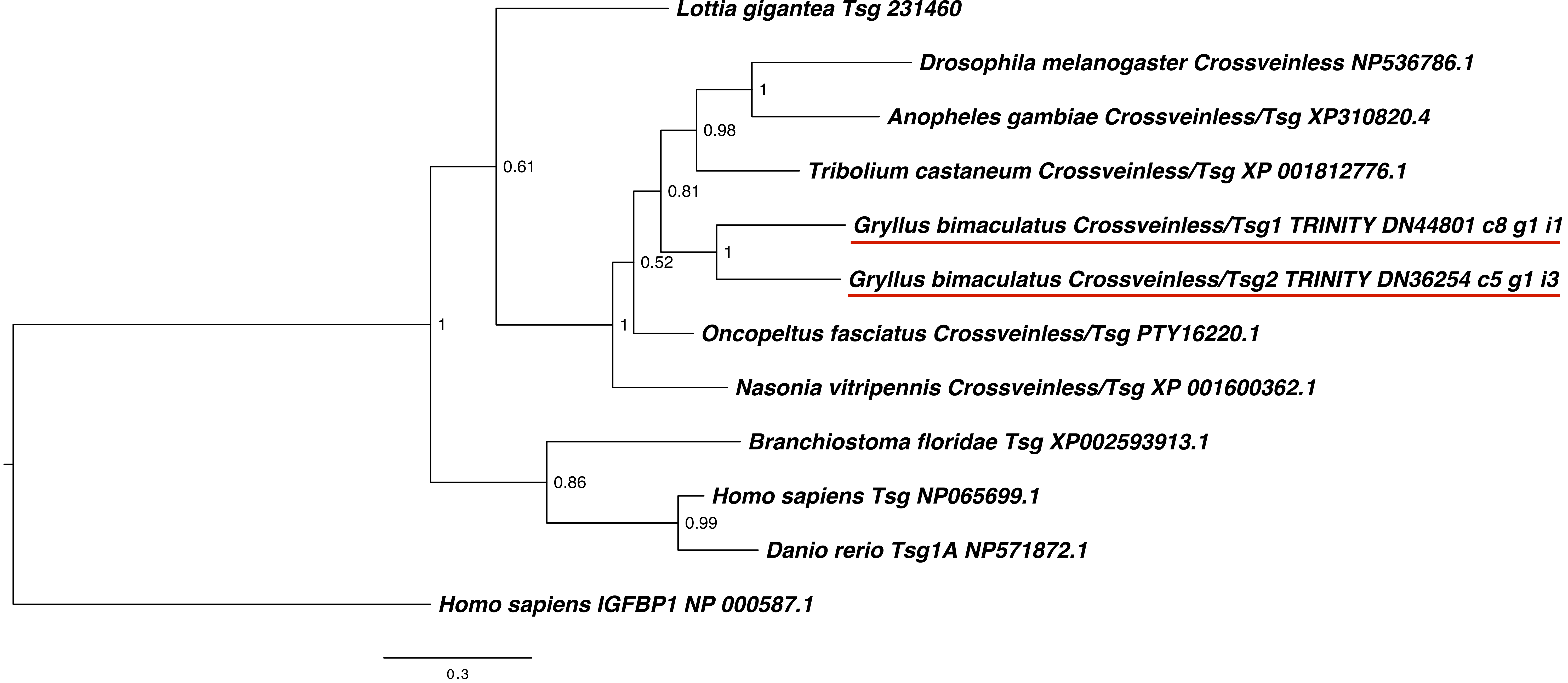

Lottia gigantea Tsg 231460
Drosophila melanogaster Crossveinless NP536786.1
Anopheles gambiae Crossveinless/Tsg XP310820.4
Tribolium castaneum Crossveinless/Tsg XP 001812776.1
Gryllus bimaculatus Crossveinless/Tsg1 TRINITY DN44801 c8 g1 i1
Gryllus bimaculatus Crossveinless/Tsg2 TRINITY DN36254 c5 g1 i3
Oncopeltus fasciatus Crossveinless/Tsg PTY16220.1
Nasonia vitripennis Crossveinless/Tsg XP 001600362.1
Branchiostoma floridae Tsg XP002593913.1
Homo sapiens Tsg NP065699.1
Danio rerio Tsg1A NP571872.1
Homo sapiens IGFBP1 NP 000587.1
0.61
1
0.98
0.81
1
0.52
1
1
0.86
0.99
0.3
