# Supplementary material for: Striking parallels between dorsoventral patterning in Drosophila and Gryllus reveal a complex evolutionary history behind a model gene regulatory network
Source: eLife. 2021 Mar 30;10:e68287. doi: 10.7554/eLife.68287 (PMC8051952; doi:10.7554/eLife.68287)
Supplement: Supplementary file 3. [file elife-68287-supp3.zip › File S3 /TreeFigures/Windbeutel.pdf]

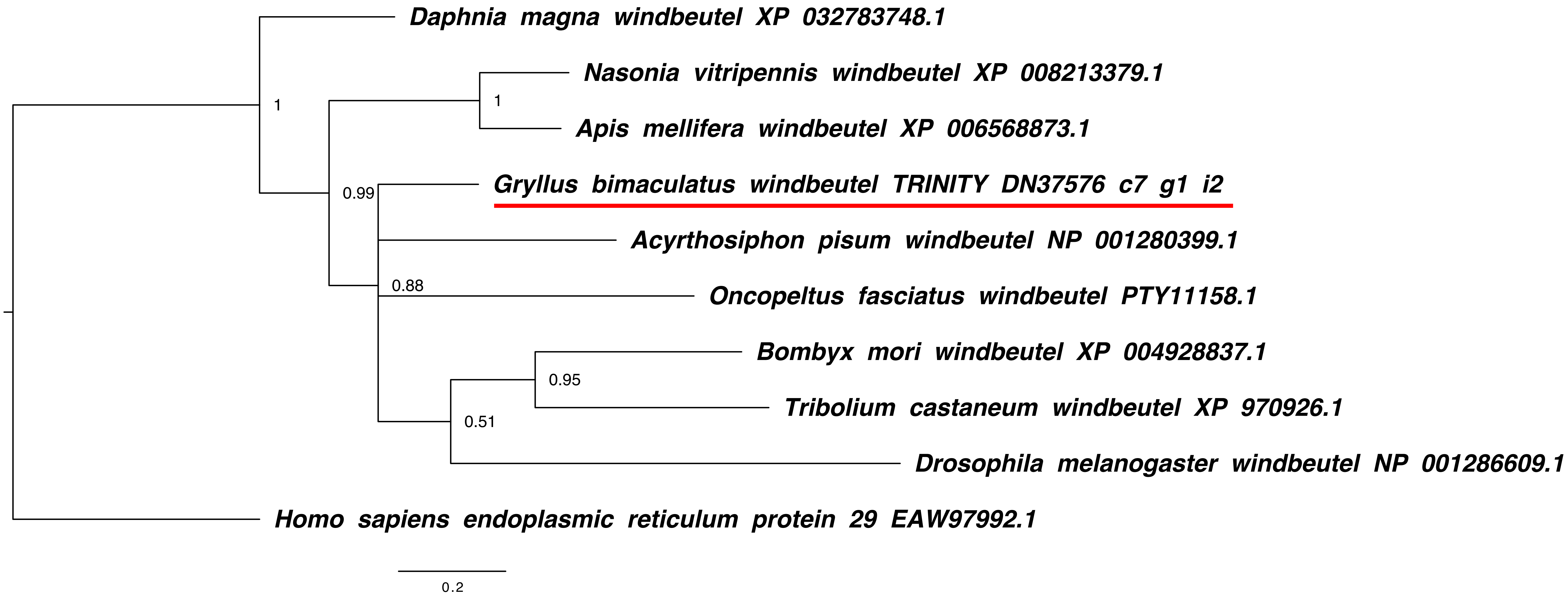
Daphnia magna windbeutel XP 032783748.1
Nasonia vitripennis windbeutel XP 008213379.1
Apis mellifera windbeutel XP 006568873.1
Gryllus bimaculatus windbeutel TRINITY DN37576 c7 g1 i2
Acyrthosiphon pisum windbeutel NP 001280399.1
Oncopeltus fasciatus windbeutel PTY11158.1
Bombyx mori windbeutel XP 004928837.1
Tribolium castaneum windbeutel XP 970926.1
Drosophila melanogaster windbeutel NP 001286609.1
Homo sapiens endoplasmic reticulum protein 29 EAW97992.1
1
1
0.99
0.88
0.95
0.51
0.2
